# Repurposing anti-phage defenses to differentially arrest the viral lifecycle reveals the regulatory logic of a parasitic satellite

**DOI:** 10.1128/msystems.00428-26

**Published:** 2026-09-02

**Authors:** S. Tansu Bagdatli, Kimberley D. Seed

**Affiliations:** 1 Department of Plant and Microbial Biology, University of California, Berkeley 1438, Berkeley, California, USA; University of Wisconsin-Madison, Madison, Wisconsin, USA

**Keywords:** bacteriophages, satellites, transcription, phage defense, *Vibrio cholerae*

## Abstract

Mobile genetic elements frequently encode defense mechanisms to protect their bacterial hosts from viral attack. In *Vibrio cholerae*, these defensive elements include phage-inducible chromosomal island-like elements (PLEs), which are phage satellites that act as highly specialized parasites of the lytic phage ICP1. While PLE transcriptional activation upon ICP1 infection is known to be temporally regulated, the underlying regulatory logic and dependencies on the progression of the phage’s developmental program required for activation remain unclear. In this study, we took a novel approach to define these dependencies by introducing independent anti-phage defense systems, BREX and DarTG, as molecular roadblocks to impede the ICP1 lifecycle. We discovered that, for both ICP1 and PLE, late-stage gene expression is fundamentally uncoupled from genome replication, representing a striking departure from the standard paradigm for double-stranded DNA phages. While BREX restricts ICP1 to an immediate-early transcriptional state that stalls PLE activation, DarTG allows the phage to execute its full transcriptional cascade despite the total block in DNA replication. This permissive environment provides the necessary cue(s) for complete PLE induction, revealing that robust PLE activation is profoundly dependent on the transcriptional progression of its helper phage.

**IMPORTANCE:**

Bacteria and their viruses (phages) are locked in perpetual evolutionary conflict. Some bacteria harbor phage satellites, specialized parasites that are activated to hijack the phage’s components to spread all the while inhibiting viral production. While some satellites respond to a single viral trigger, the regulation of many satellites, including clinically relevant phage-inducible chromosomal island-like elements (PLEs) in *Vibrio cholerae*, remains poorly understood. Here, we used bacterial defense systems as molecular roadblocks to probe how PLE activation depends on its helper phage. We found that severe disruptions to viral transcription stall PLE activation. Unexpectedly, both the virus and the satellite can execute their full transcriptional programs even when DNA replication is completely blocked, challenging a fundamental paradigm in virology. These insights reveal a sophisticated level of phage-satellite coordination, illustrating how satellite activation is tightly linked to the transcriptional state of its helper phage, a dependency that ultimately drives the dissemination of mobile genetic elements.

## INTRODUCTION

Phages, the viruses that infect bacteria, shape bacterial population dynamics by infecting, manipulating, and killing their bacterial hosts. This dynamic interaction exerts intense selective pressure, driving bacteria to evolve a diverse arsenal of anti-phage defense systems (1). These systems are frequently encoded by mobile genetic elements (MGEs) (2) and can be broadly categorized by the timing and underlying mechanism of their action. Early-acting, cell-autonomous systems, such as restriction-modification systems and bacteriophage exclusion (BREX), distinguish self from non-self DNA, often preventing phage DNA replication immediately upon entry (3–5). In contrast, abortive infection (Abi) systems may be triggered later and function as a communal defense, in which the infected cell dies upon phage detection, preventing phage proliferation and safeguarding the surrounding bacterial population (1, 6). The DarTG toxin-antitoxin system exemplifies this strategy, whereby the DarT toxin chemically modifies phage DNA to halt its replication, triggering cell death that protects uninfected neighbors (7).

While systems like DarTG are standalone defenses, some MGEs have evolved to function as highly specialized Abi systems that parasitize invading phages. In *Vibrio cholerae*, the agent of cholera (8), these elements are a distinct family of phage satellites known as phage-inducible chromosomal island-like elements (PLEs). *V. cholerae* is persistently attacked by the lytic myovirus ICP1 during human infection (9, 10), and PLEs provide highly specific defense against ICP1 by acting as both selfish phage parasites and effective abortive infection systems (10, 11). The PLE life cycle is tightly coupled to the ICP1 life cycle. Upon ICP1 infection, PLE is transcriptionally activated and executes a temporally regulated gene expression cascade comprising early, middle, and late phases that mirrors the phage’s own rapid 20-min infection cycle (12). During this time, PLE excises from the *V. cholerae* chromosome (13), replicates to high copy numbers (14), and hijacks ICP1 structural proteins to package its own genome into PLE transducing particles (15), while simultaneously abolishing progeny production by blocking late-stage phage morphogenesis (15–17).

As phage satellites, PLEs belong to a widespread class of MGEs defined by their parasitism of a helper phage to promote their own mobilization (18). The canonical model for how phage satellites sense and respond to their helper is based on the *Staphylococcus aureus* pathogenicity islands (SaPIs). In the absence of helper phage, SaPIs are maintained in a dormant state by a master repressor; upon infection, a single phage protein acts as an anti-repressor, triggering satellite activation (19, 20). However, PLE activation appears to deviate from this canonical single-trigger model. Unlike SaPI helper phages, which can escape satellite induction via point mutations in the non-essential anti-repressor (20), spontaneous ICP1 mutants that can escape PLE-mediated restriction have not been reported. This absence of escape mutants suggests that PLE likely responds to an essential phage protein that is intolerant of mutation, or that it relies on functionally redundant helper cues to ensure its activation. While we have previously established that the bacterial protein H-NS silences the model variant PLE1 in the absence of infection (21), the precise viral cues that trigger PLE activation remain unknown.

Given the absence of an apparent single SaPI-like activation trigger, we hypothesized that the transcriptional regulation of PLE is coupled to the progression of the ICP1 life cycle. To interrogate these dependencies, we used BREX and DarTG defense systems as distinct molecular tools to limit the progression of ICP1’s life cycle. Both systems block ICP1 genome replication (22, 23), but they represent distinct early-acting and abortive strategies. Critically, it was unknown how these different defenses impact the progression of the phage’s temporal transcriptional cascade. We reasoned that despite their shared inhibition of phage genome replication, these systems might serve as temporal roadblocks at different stages of the phage’s developmental program. By characterizing these barriers, we aimed to decouple phage replication from transcriptional progression. This approach allowed us to determine the extent to which PLE activation depends on the unimpeded progression of the phage transcriptional program, providing critical insight into the underlying regulatory logic that governs this satellite.

By employing this comparative approach, we uncovered a critical divergence in how BREX and DarTG defenses impact phage development. While both block ICP1 genome replication (22, 23), we found they impose drastically different constraints on phage gene expression. In the presence of BREX, phage gene expression is potently repressed. In contrast, DarTG—despite blocking replication—allows the phage to progress to late-stage gene expression. This finding demonstrates that for ICP1, the activation of late genes encoding structural and lysis proteins occurs independently of successful genome replication—a striking deviation from the standard paradigm for double-stranded DNA viruses, including phages (24, 25). We leveraged these contrasting transcriptional landscapes to probe the requirements for PLE activation and found that PLE gene expression is strictly dependent on the progression of the phage’s transcriptional program. In the presence of BREX, PLE gene expression is severely attenuated and varies by operon, whereas in the DarTG background, PLE executes its full transcriptional activation program. Notably, this reveals that PLE mirrors the phage’s capacity to uncouple transcriptional progression from genome replication, as the satellite activates late gene expression even when its genome replication is blocked. Collectively, our findings demonstrate that robust PLE activation is intimately tied to the global transcriptional state of its helper phage. By mechanically stalling the viral life cycle, we reveal that complete PLE induction requires uninterrupted phage gene expression, suggesting that activation depends on phage-derived triggering factor(s) reaching a critical expression threshold.

## RESULTS

### Comparative transcriptomics reveals distinct global impacts of BREX and DarTG on PLE and phage gene expression

**Fig 1:**
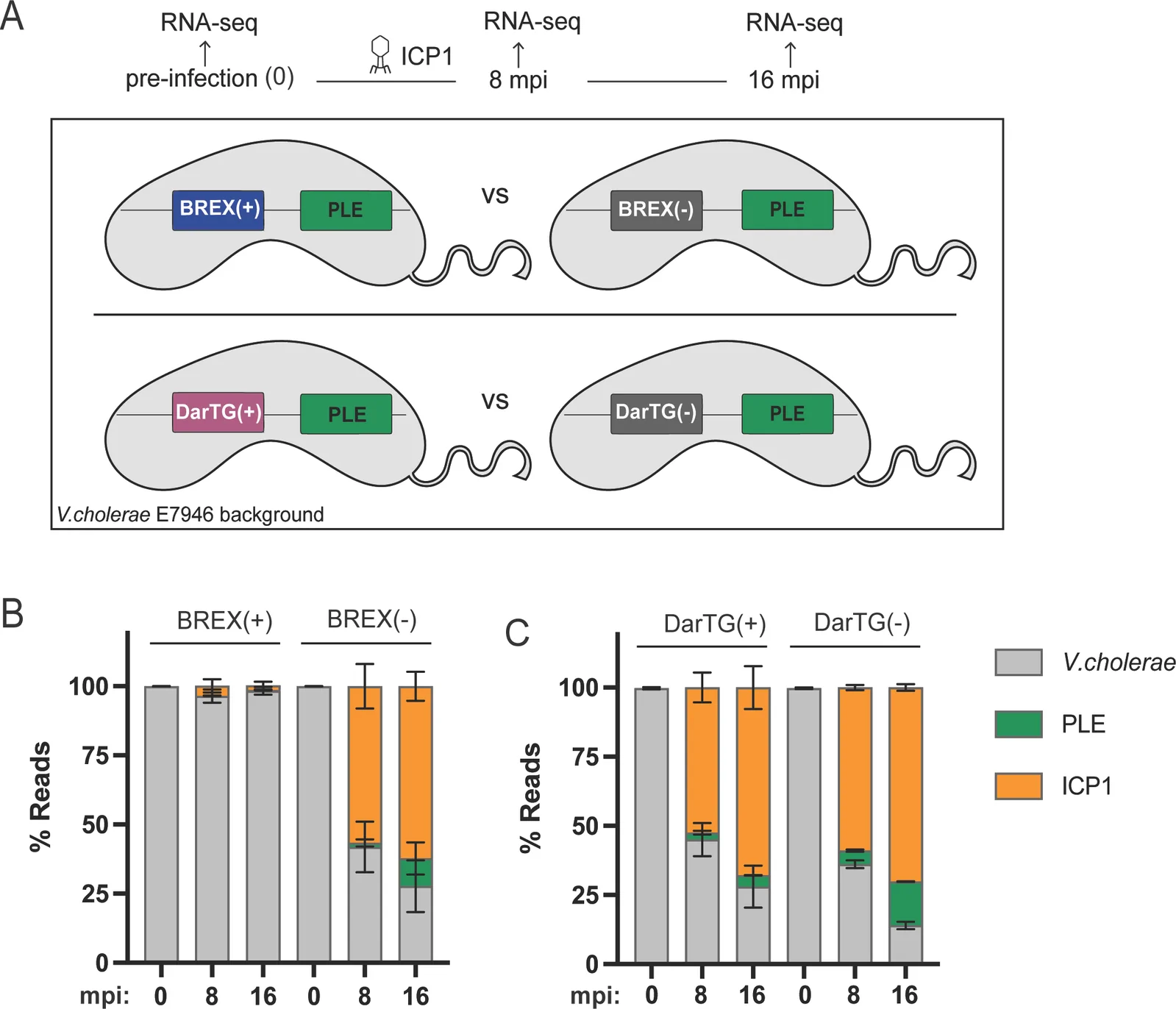
Experimental framework to evaluate the transcriptional impact of defense systems on phage and satellite gene expression. (**A**) Schematic of the RNA-seq experimental design. Samples were collected from *V. cholerae* E7946 PLE(+) strains before ICP1 infection (*t* = 0), and at 8 and 16 min post-infection (mpi). Isogenic strain sets were compared to evaluate the impact of functional BREX and DarTG defenses (+) against their respective replication-permissive controls (−). (**B**) Proportional transcript abundance (percent reads) of the *V. cholerae* host, PLE, and ICP1 over the infection time course in the presence and absence of BREX. (**C**) Proportional transcript abundance (percent reads) of the host, PLE, and ICP1 over the infection time course in the presence and absence of DarTG. For both panels, the bar height represents the mean of three biological replicates; error bars indicate standard deviation.

We previously identified two anti-phage defense systems that block the initiation of ICP1 genome replication in clinical isolates of *V. cholerae*: BREX, encoded by an integrative and conjugative element (22), and DarTG, a toxin-antitoxin system encoded by a distinct phage defense element (23). To enable appropriate comparisons, we separately introduced the genetic elements encoding BREX and DarTG into the same parental *V. cholerae* strain harboring the most well-studied PLE variant, PLE1 (hereafter PLE) (Fig. 1A). We then generated isogenic controls by deleting the genes responsible for inhibiting ICP1 replication within each element (see methods). This process yielded two matched sets of strains: functional defense [BREX(+) and DarTG(+)] and replication-permissive controls [BREX(−) and DarTG(−)]. This design allows us to compare infection restricted by distinct defenses, both of which inhibit ICP1 genome replication (Fig. S1A), with one in which the phage developmental program proceeds. Although PLE ultimately provides abortive defense by blocking late-stage phage assembly (15–17), it exerts minimal impact on ICP1 genome replication and transcription (12, 14). Consequently, the defense-deficient strains serve as replication-permissive controls, allowing us to assess the specific transcriptional constraints imposed by BREX and DarTG.

To understand how these defense systems impact phage and satellite gene expression, we performed RNA sequencing (RNA-seq) on samples collected immediately before infection (0), during active viral replication in a productive infection (8 min), and late in the infection cycle (16 min) (12) (Fig. 1A). Principal component analysis confirmed distinct clustering by infection condition and genetic background (Fig. S1B). In the absence of BREX, we observed rapid transcriptional takeover by the phage and satellite: by 16 min post-infection (mpi), ICP1 and PLE transcripts constituted 62% and 11% of the total reads, respectively (Fig. 1B), closely mirroring previous analyses done in the same strain background without any additional defense-associated MGE (12). On the other hand, the presence of BREX severely limited this takeover, with host transcripts remaining dominant throughout the time course and ICP1 and PLE combined accounting for just 2% of total reads at 16 mpi (Fig. 1B). The absence of phage transcriptional takeover is consistent with the ability of BREX to block phage production while permitting cell survival (3). In stark contrast, DarTG did not repress the global accumulation of phage transcripts. At 16 mpi, ICP1 transcripts dominated the population equally in the presence or absence of the defense (67% and 70% of total reads, respectively; unpaired *t*-test *P* = 0.625) (Fig. 1C). However, while phage transcription was largely unaffected, PLE transcripts were significantly reduced in the presence of DarTG, accounting for only 4% of total reads compared to the 15% in the DarTG(−) control, (unpaired *t*-test *P* < 0.0001) (Fig. 1C).

### BREX potently arrests ICP1 gene expression, while DarTG permits replication-independent late gene expression

**Fig 2:**
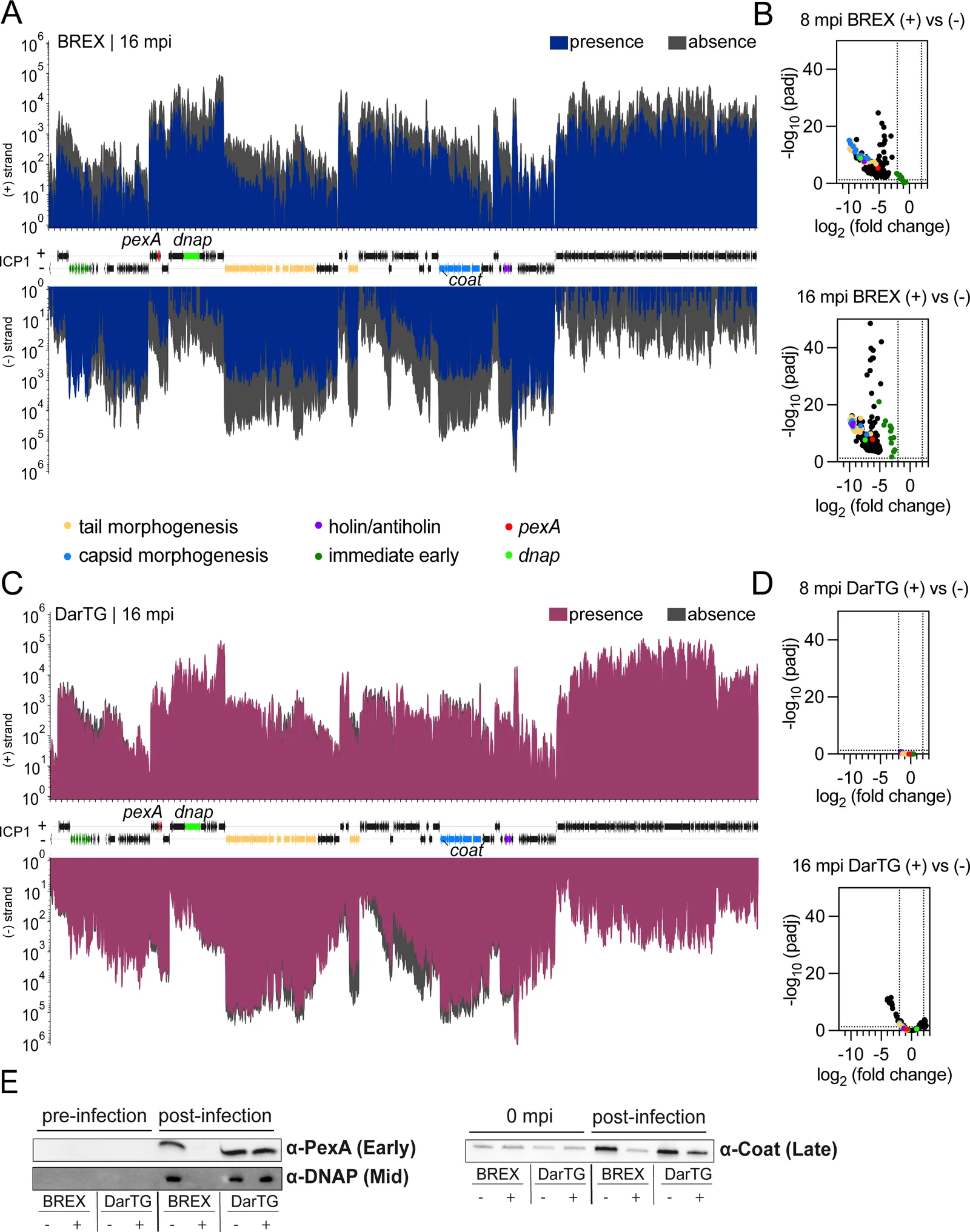
ICP1 gene expression progression is altered in the presence of anti-phage elements. (**A**) Average RNA-seq read coverage mapped to the ICP1 genome at 16 mpi in the presence or absence of BREX. Forward strand (+) reads are plotted above the *x*-axis and reverse strand (–) reads below the *x*-axis. Coverage represents the mean of three biological replicates and is displayed on a log-scaled *y*-axis. For panels A–D, ICP1 gene features are colored based on known or predicted functions according to the legend below panel A, while genes without functional annotation are shown in black. (**B**) Volcano plot illustrating the differential expression of ICP1 genes in the presence vs absence of BREX at 8 and 16 mpi. Vertical dotted lines indicate a log_2_ fold change cutoff of ≥±2, and horizontal dotted lines indicate a significance threshold of −log_10_ (*P*_adj_) ≥1.3. (**C**) Strand-specific RNA-seq coverage mapped to the ICP1 genome at 16 mpi in the presence or absence of DarTG. Plotting conventions are the same as in panel A. (**D**) Volcano plot illustrating the differential expression of ICP1 genes in the presence vs absence of DarTG at 8 and 16 mpi. Cutoff lines are the same as in panel B. (**E**) Western blots of ICP1 proteins probed in cells with and without BREX or DarTG. For the detection of PexA (early), DNAP (mid), and coat (late) proteins, samples were assessed at 8, 12, and 16 mpi, respectively. For the coat protein, the faint band appearing in infected BREX(+) cells originates from the initial phage inoculum. Samples at 0 mpi, (immediately upon addition of the phage inoculum), were probed to demonstrate that this faint band is a structural component of the input phage rather than *de novo* synthesis. The images are representative of three biological replicates. For the uncropped images of all replicates and quantification, see Fig. S3 and S4.

We next examined how these defenses alter the temporal progression of the phage life cycle. In the presence of BREX, ICP1 transcription was severely impaired. Visualization of coverage tracks revealed a global reduction in reads mapping to the phage genome compared to the permissive background (Fig. 2A; Fig. S2A). Differential expression analysis at 16 mpi showed that all phage transcripts were significantly downregulated (Fig. 2B; Table S2). Analyses at both 8 and 16 mpi showed that this repression was most dramatic for the late-gene operons encoding capsid (*gp120-128*), and tail (*gp69-87* and *gp91-94*) morphogenesis proteins, as well as the holin/antiholin (*gp137-138*) lysis module (Fig. 2B). Interestingly, at 8 mpi, a small subset of immediate early genes (11 out of 227 genes) was not differentially regulated in the BREX(+) background. (Fig. 2B; Table S1). This suggests that while BREX permits the initiation of the phage developmental program, it causes a premature arrest shortly after the onset of early transcription.

In stark contrast, visualization of coverage tracks revealed that DarTG had a minimal impact on the phage transcriptional program throughout the infection (Fig. 2C; Fig. S2B). At 8 mpi, no ICP1 genes were differentially regulated (Fig. 2D; Table S3). By 16 mpi, only a small subset of the phage genome (12 out of 227 genes, all of unknown function) was significantly downregulated (Fig. 2D; Table S4). The vast majority of the ICP1 transcriptome remained unaffected, indicating that, unlike BREX, DarTG allows the phage to execute a nearly complete temporal gene expression cascade despite blocking genome replication.

To determine if these transcriptional profiles were reflected at the protein level, we performed western blots for representative early (PexA), middle (DNA polymerase, DNAP), and late (coat) phage proteins. Consistent with the severe repression observed by RNA-seq, no *de novo* synthesis of these proteins was detectable in the presence of BREX (Fig. 2E). This demonstrates that BREX, whose mechanism of defense remains unknown, restricts phage gene expression. Conversely, in the DarTG(+) background, all three phage proteins were readily detectable, confirming that the phage progresses to late-stage gene expression despite the replication block (Fig. 2E). However, densitometric quantification revealed nuances in the efficiency of this progression. While early and middle protein levels (PexA and DNAP, respectively) remained comparable to the replication-permissive control (Fig. S3A and S4A), we observed a significant, approximately 2-fold reduction in the accumulation of the coat protein in the presence of DarTG (Fig. S3B). This protein-level decrease is consistent with a downward trend in coat (*gp122*) transcription observed in our RNA-seq data [log_2_ fold change = −1.5 compared to DarTG(−)]. However, this transcriptomic change did not meet the stringent significance thresholds required for global differential expression analysis.

Together, these results reveal that while both defense systems block phage genome replication, they impose vastly different constraints on phage gene expression. BREX potently arrests ICP1 gene expression shortly after entry. In contrast, DarTG allows the phage to produce late-stage structural proteins even in the absence of a replicated genome. This disparate effect also reveals a surprising uncoupling of late gene transcription from genome replication in ICP1, a finding that deviates from the canonical model for other phages (24) and some dsDNA eukaryotic viruses (25), in which viral genome replication is a prerequisite to license the activation of late gene expression.

### PLE gene expression is uncoupled from its genome replication

**Fig 3:**
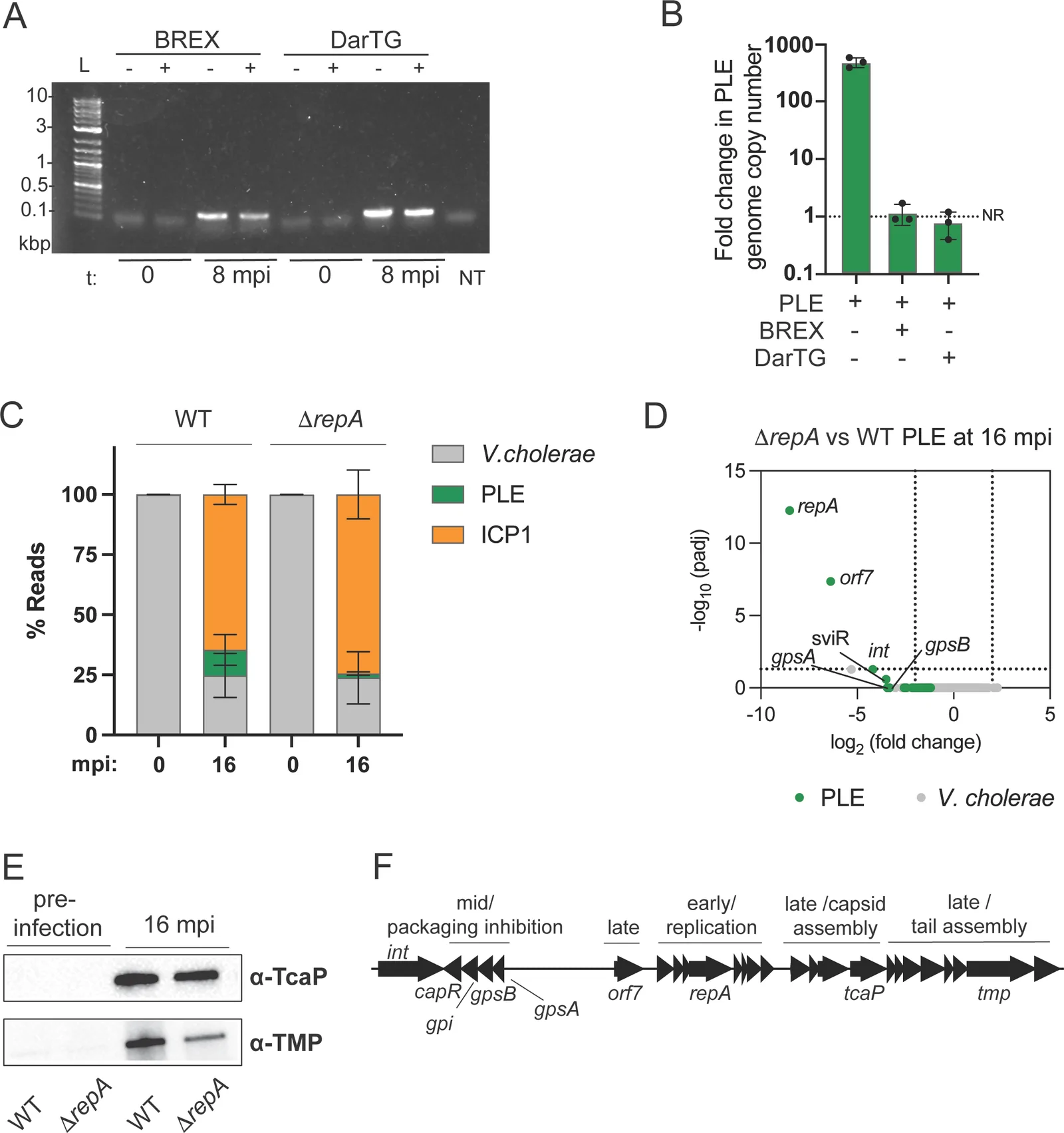
PLE transcriptional activation is uncoupled from genome replication. (**A**) PCR analysis of PLE circularization before (*t* = 0) and 8 mpi in the presence (+) or absence (−) of the indicated defense system. L, ladder; NT, no template control. For additional biological replicates, see Fig. S5A. (**B**) Fold change in PLE genome copy number as assessed by qPCR at 20 mpi by ICP1. NR, no replication. (**C**) Proportional transcript abundance (percent reads) of the *V. cholerae* host, PLE, and ICP1 before (0) and 16 mpi in WT PLE and the replication-deficient Δ*repA* mutant. (**D**) Volcano plot illustrating differential gene expression of Δ*repA* vs WT backgrounds at 16 mpi. Vertical dotted lines indicate a log_2_ fold change cutoff of ≥±2, and horizontal dotted lines indicate a significance threshold of −log_10_ (*P*_adj_) ≥1.3. (**E**) Representative western blots for PLE-encoded late proteins TcaP and TMP in WT and Δ*repA* backgrounds before (pre-infection) and 16 mpi by ICP1. For the uncropped images of all replicates and quantification, see Fig. S5B through D. (**F**) Schematic of the PLE1 genome. Genes are organized into temporal expression blocks (early, mid, and late) based on peak expression during ICP1 infection (12), with corresponding functional modules indicated above.

Given the divergent effects of BREX and DarTG on the helper phage’s gene expression, we hypothesized that these defenses would impact the PLE life cycle in distinct ways. We first examined PLE excision and circularization, the initial activation step triggered by the ICP1-encoded protein PexA (13). We found that despite the presence of either BREX or DarTG defense systems, PLE successfully excises from the *V. cholerae* chromosome (Fig. 3A). While successful PLE excision was expected in the presence of DarTG, where PexA is expressed, it was surprising in the BREX(+) background, given that PexA was undetectable by western blot (Fig. 2E). This suggests that the threshold of PexA required to stimulate PLE excision is extremely low and falls below the limit of detection in our western blot analysis.

Following excision, PLE replicates to high copy number, a process that remains incompletely understood but is known to require the PLE-encoded replication initiation factor, RepA (14), and an ICP1-encoded helicase (26), and likely ICP1-encoded DNAP (14). With this in mind, we next measured the fold change in PLE copy number by qPCR and found that PLE replication is completely blocked in the presence of BREX or DarTG (Fig. 3B). This observation prompted us to determine if PLE’s transcriptional activation is dependent on its own genome replication, as a block in this process could, in turn, be responsible for any observed effects on gene expression.

To directly test if PLE replication is a prerequisite for its transcriptional activation, we compared the transcriptional profiles of wild-type (WT) PLE and the replication-deficient mutant (Δ*repA*) at 16 mpi by RNA-seq. Strikingly, principal component analysis showed that WT and Δ*repA* conditions cluster similarly during infection (Fig. S1B). In the WT background, PLE reads accounted for 10.6% of the total transcriptome, whereas in the Δ*repA* mutant, PLE transcripts accounted for 1.7% (unpaired *t*-test *P* = 0.134)*,* a comparably high level of expression compared to the 0.03% observed in uninfected cells (Fig. 3C). Differential gene expression analysis confirmed only *orf7* was significantly downregulated in the Δ*repA* mutant (Fig. 3D). While a subset of the remainder of the PLE genome (Fig. 3F)—including the integrase (*int*)*,* the packaging inhibition operon (*gpsAB*, *gpi*, and *capR*) (16), and the small non-coding RNA SviR (27)—exhibited a trend toward lower expression in the Δ*repA* mutant (with log_2_ fold change <−3), they did not meet the stringent significance thresholds required to be defined as significantly downregulated (Fig. 3D; Table S5).

To validate this observed transcriptional activation of PLE at the protein level, we assessed the production of TcaP (15) and the tape measure protein (TMP) (27), two late-stage (12) PLE-encoded structural proteins involved in the formation of PLE transducing particles. Consistent with the RNA-seq data, both proteins were produced even in the absence of PLE replication (Fig. 3E). However, while TcaP levels were comparable to WT, TMP abundance was significantly reduced in the Δ*repA* mutant (Fig. S5B through D). Since *tmp* (*orf22*) transcripts were not significantly downregulated (Table S5), this reduction likely reflects post-transcriptional regulation, potentially linked to the lower abundance of SviR. SviR is known to increase the transcript stability of the *capR* operon (28); correspondingly, this operon also trended toward lower expression in the Δ*repA* mutant (log_2_ fold change −3.3). Altogether, these data demonstrate that PLE’s robust transcriptional activation during ICP1 infection—including the production of late-stage structural proteins—occurs independently of its genome replication. This aligns with our previous findings that PLE replication is not required for the functional inhibition of ICP1 (14, 26).

### BREX stalls PLE gene expression

We reasoned that because the anti-phage defense systems BREX and DarTG disrupt the progression of ICP1 gene expression to different extents, they could serve as tools to probe how PLE’s transcriptional program responds to perturbations in the ICP1 life cycle. Since we established that PLE replication is not necessary for its transcriptional activation (Fig. 3), we investigated the transcriptional activity of PLE in the presence and absence of BREX in greater detail.

**Fig 4:**
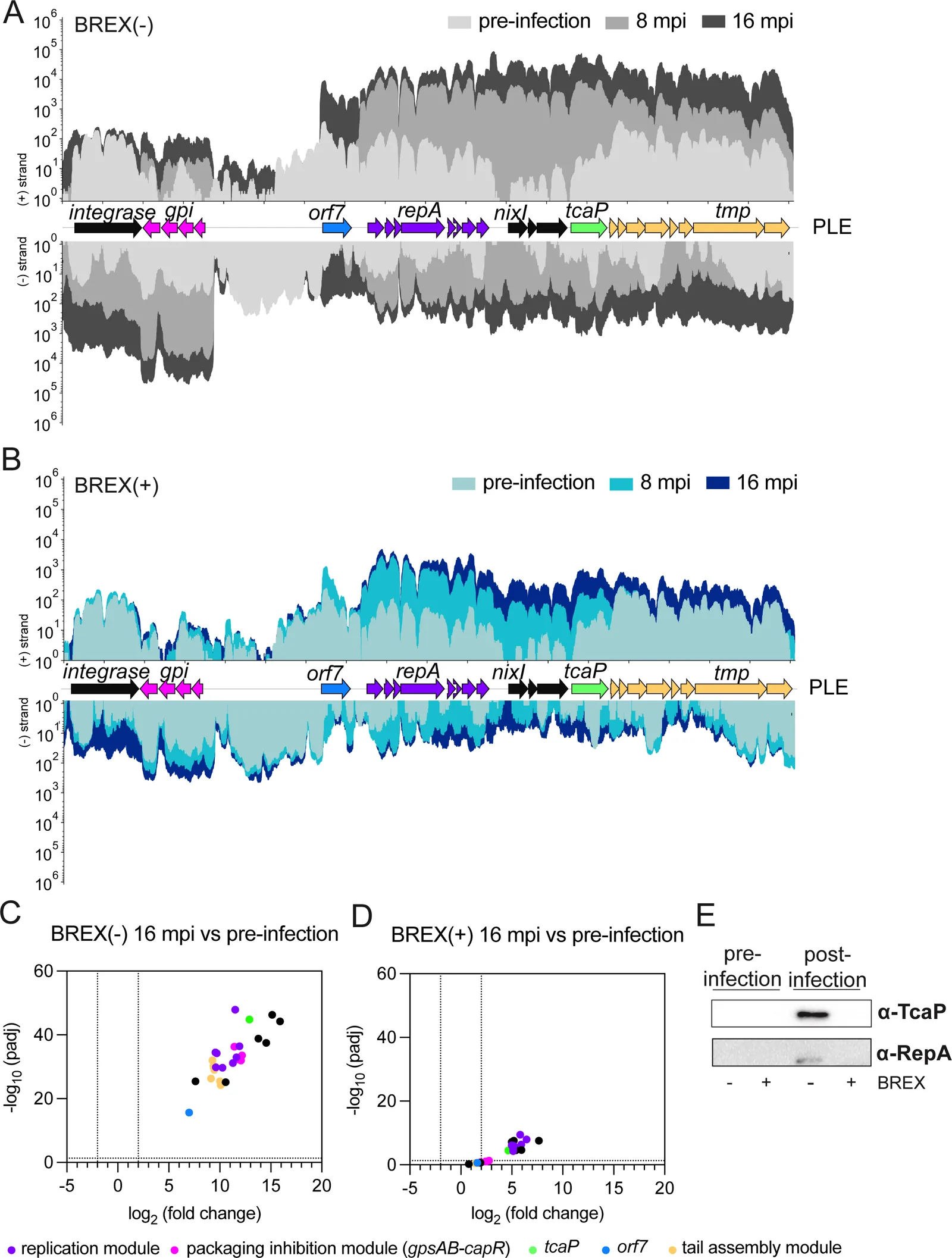
BREX stalls the PLE transcriptional program. (**A** and** B**) Average RNA-seq read coverage mapped to the PLE genome in (**A**) BREX(−) and (**B**) BREX(+) cells. Coverage tracks represent uninfected, 8 mpi, and 16 mpi time points. Forward-strand reads are plotted above the *x*-axis and reverse-strand reads below. Coverage represents the mean of three biological replicates (with the exception of two biological replicates for BREX(+) pre-infection sample, see methods for detail) and is displayed on a log-scaled *y*-axis. (**C **and **D**) Volcano plots illustrating differential PLE gene expression at 16 mpi compared to pre-infection in (**C**) BREX(−) and (**D**) BREX(+) backgrounds. Each point represents a PLE gene. Selected genes belonging to the functional modules defined in Fig. 3F are colored according to the legend, while other PLE genes are shown in black. Vertical dotted lines indicate a log_2_ fold change cutoff of ≥±2, and horizontal dotted lines indicate a significance threshold of −log_10_ (*P*_adj_) ≥1.3. (**E**) Western blots for PLE-encoded TcaP (late) and RepA (early) probed in cells with (+) and without (−) BREX before and after infection. TcaP was probed at 16 mpi and RepA at 12 mpi. Images are representative of three biological replicates. For the uncropped images of all replicates, see Fig. S7.

First, we examined the read coverage plots of PLE in the BREX(−) background. In the absence of BREX, read coverage plots demonstrate that, as expected, the entire PLE transcriptional program was robustly induced upon ICP1 infection (Fig. 4A). Differential expression analysis revealed that all PLE genes were upregulated at 8 and 16 mpi compared to uninfected cells, with expression levels increasing as the infection progressed (Fig. 4C; Fig. S6A; Tables S6 and S7).

In stark contrast, read coverage plots of PLE in the BREX(+) background showed that gene expression remained minimal throughout the course of infection (Fig. 4B). However, differential expression analysis comparing infected to uninfected BREX(+) cells revealed that a subset of genes, comprising the early replication operon (*orf8*–*orf14*) and *nixI-tcaP*, is significantly upregulated during infection (Fig. 4D; Fig. S6A; Table S8 and S9). While the magnitude of this induction is lower than in the BREX(−) background, the initial activation of the early operon is clearly discernible. For example, at 8 mpi, *repA* transcript levels increased 22-fold in BREX(+) cells compared to a ~330-fold increase in the BREX(−) control. As the infection progresses, this developmental stall is exacerbated; by 16 mpi, *repA* and *tcaP* showed only 35- and 25-fold increases in BREX(+) cells, respectively, compared to >1,000- and >7,000-fold increases in the BREX(−) background. Crucially, the remaining PLE modules, including the packaging inhibition operon (*gpsAB* and *gpi*), *capR*, *orf7*, and the tail assembly operon (*orf18*–*orf23*), did not show any significant upregulation in the presence of BREX. This selective, low-level activation could point to a multi-cue licensing model, in which immediate-early phage input(s) trigger the PLE early operon, but downstream modules remain silent because the arrested phage lifecycle fails to reach the specific milestones required to activate them. Alternatively, the data are consistent with a model in which trace levels of a specific phage-derived triggering factor do not reach the threshold necessary for complete and robust transcriptional activation of the satellite.

To validate these findings at the protein level, we assessed the production of the PLE-encoded early protein RepA and the late protein TcaP. Strikingly, despite the significant >20-fold increase in transcript levels observed for both genes, neither protein was detectable by western blot in the presence of BREX (Fig. 4E). This reveals a notable disconnect between transcript accumulation and protein production. While the absolute abundance of these transcripts in the BREX(+) background may remain below a required threshold for robust translation, particularly when compared to the substantially higher induction seen in the BREX(−) background, this result suggests that BREX may impose an additional block on gene expression post-transcriptionally. Alternatively, the premature arrest of the ICP1 life cycle by BREX may deprive PLE of specific helper-encoded factors required for efficient translation or stability of its own proteins. Regardless of the mechanism, the absence of RepA directly aligns with the complete block in PLE replication observed in these cells (Fig. 3B). Overall, these results demonstrate that while PLE can initiate the first steps of its transcriptional program, BREX effectively stalls the satellite life cycle before proteins can be produced.

### DarTG permits robust PLE gene expression

**Fig 5:**
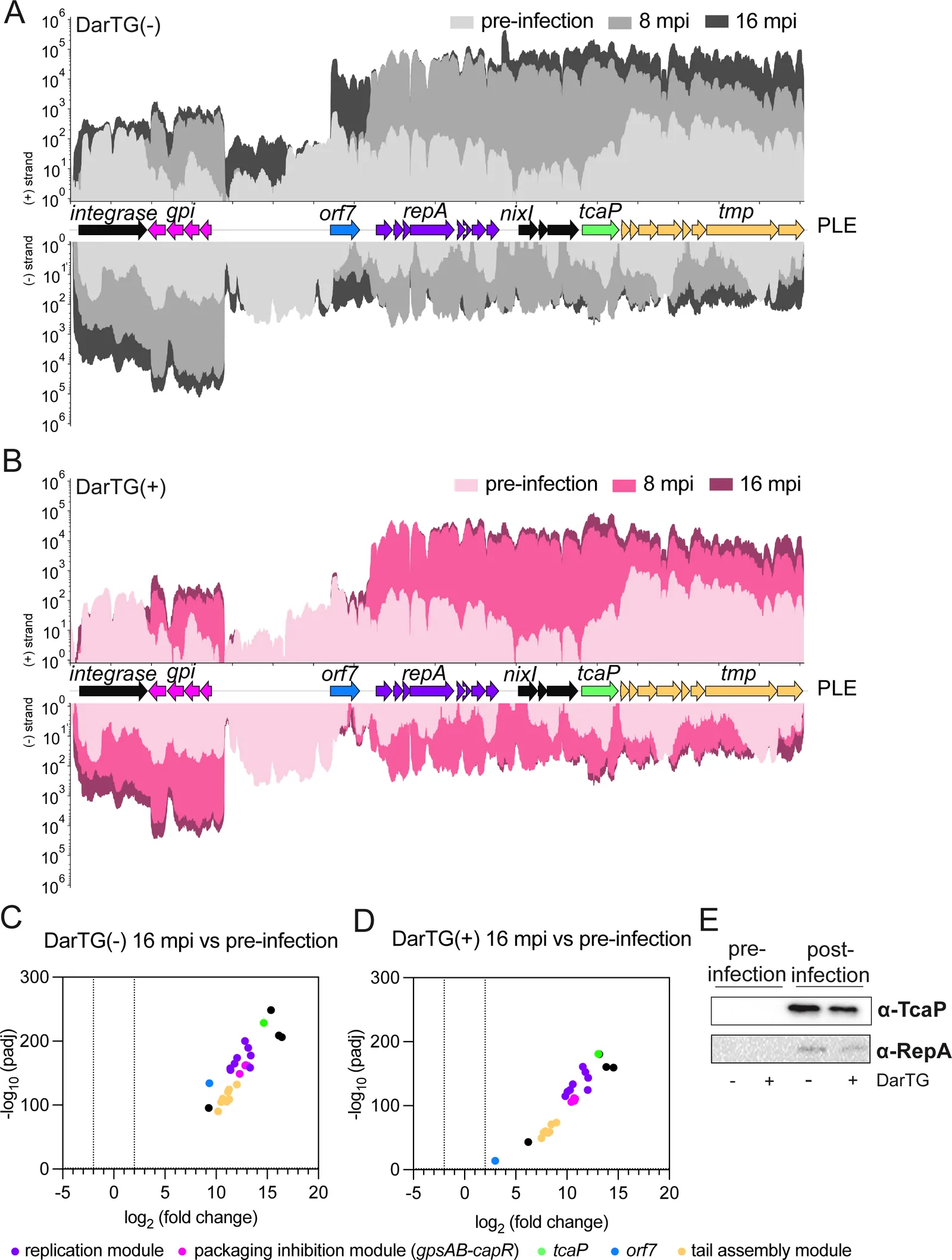
DarTG permits the progression of the PLE transcriptional program. (**A** and** B**) Average RNA-seq read coverage mapped to the PLE genome in (**A**) DarTG(−) and (**B**) DarTG(+) cells. Coverage tracks represent uninfected, 8 mpi, and 16 mpi time points. Forward-strand reads are plotted above the *x*-axis and reverse-strand reads below. Coverage represents the mean of three biological replicates and is displayed on a log-scaled *y*-axis. (**C **and **D**) Volcano plots illustrating differential PLE gene expression at 16 mpi compared to pre-infection in (**C**) DarTG(−) and (**D**) DarTG(+) cells. Each point represents a PLE gene. Selected genes belonging to the functional modules defined in Fig. 3F are colored according to the legend, while other PLE genes are shown in black. Vertical dotted lines indicate a log_2_ fold change cutoff of ≥±2, and horizontal dotted lines indicate a significance threshold of −log_10_ (*P*_adj_) ≥1.3. (**E**) Western blots for PLE-encoded TcaP (late) and RepA (early) probed in cells with (+) and without (−) DarTG before and after infection. TcaP was probed at 16 mpi and RepA at 12 mpi. Images are representative of three biological replicates. For the uncropped images of all replicates, see Fig. S7.

In contrast to BREX, DarTG had a much milder effect on the overall transcriptional landscape of PLE (Fig. 5A and B). PLE is robustly activated in the presence of DarTG, despite the block in replication (Fig. 5B and 3B). Read coverage and differential expression analyses showed that the entire PLE transcriptional program was induced, although the magnitude of upregulation was slightly lower than in the DarTG(−) control (Fig. 5C and D). This observation is corroborated by the global ~4-fold reduction in PLE transcripts, from 15% to 4% of the total transcriptome (Fig. 1C), which likely reflects PLE’s replication deficiency in the presence of DarTG (Fig. 3B).

At 8 mpi, all PLE genes appeared to be upregulated to a similar magnitude in both DarTG(+) and DarTG(−) cells compared to uninfected controls (Fig. S6B; Tables S10 and S12). However, by 16 mpi, the tail morphogenesis operon (*orf18*–*orf23*) was more potently upregulated in the absence of DarTG (Fig. 5C and D; Tables S11 and S13). For example, *tmp* showed a ≈230-fold increase in the presence of DarTG, compared to a ≈2,300-fold increase in its absence. Notably, *orf7* showed the highest magnitude difference in induction, with an 8-fold increase in DarTG(+) cells compared to the >640-fold increase in the control. This specific difference can be attributed to the PLE’s inability to replicate in the presence of DarTG, as *orf7* was the only gene significantly downregulated in the Δ*repA* mutant (Fig. 3D). In contrast, at 16 mpi, PLE genes within the replication module (*orf8–orf13*) and the packaging inhibition module (*gpsAB-capR*) reached similar magnitudes of induction in both backgrounds. For example, *repA* increased ≈1,500-fold in the presence of DarTG and ≈4,200-fold in its absence (Fig. 5C and D). The observation that replication and packaging-inhibition modules remain strongly transcribed while specific operons are selectively stalled indicates that ADP-ribosylation of the DNA template does not directly interfere with general PLE transcription. Instead, this operon-specific gating suggests that full expression of PLE late genes depends on the developmental progression of the helper phage and/or the replication status of the satellite itself.

Consistent with these transcriptional profiles, PLE gene expression culminated in the production of both the early protein RepA and the late protein TcaP (Fig. 5E). These results indicate that, similar to our observations for ICP1, the production of PLE-encoded late structural proteins does not strictly depend on PLE genome replication (Fig. 5E). Although RepA protein was detectable, PLE replication remained blocked (Fig. 3B). This is consistent with the known activity of DarT, which ADP-ribosylates single-stranded DNA to inhibit DNA synthesis (29, 30). Together, these results show that, unlike the early arrest of the ICP1 life cycle in the presence of BREX, DarTG allows the ICP1 life cycle to progress sufficiently to provide the necessary permissive environment for complete PLE transcriptional activation.

## DISCUSSION

**Fig 6:**
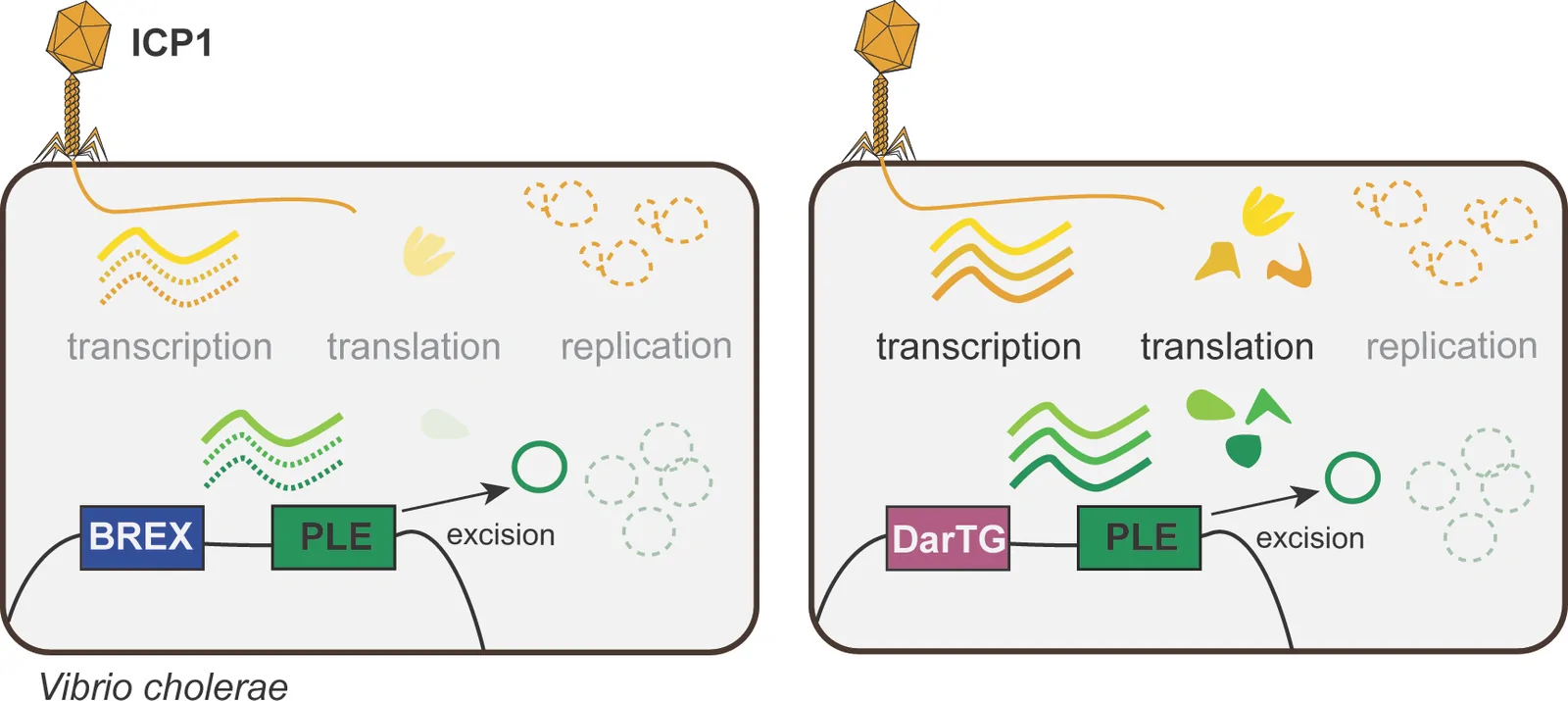
Model of ICP1 and PLE lifecycles under BREX and DarTG restriction Both defense systems completely block genome replication of both the ICP1 helper phage (orange) and the PLE satellite (green) (dashed outlines). (Left) During BREX restriction, transcription from both ICP1 and PLE is severely reduced; while some immediate early ICP1 genes (solid-light-yellow line) and early PLE genes (solid light-green line) are transcribed at or near permissive levels, downstream transcription is blocked (dotted lines). This severe transcriptional bottleneck results in a lack of detectable protein accumulation by Western blot (faint shapes). Nevertheless, sufficient levels of ICP1-encoded PexA protein are synthesized to mediate PLE excision (green ring). (Right) During DarTG restriction, temporal transcription—progressing from early to late genes (color-coded light to dark solid lines)—and translation (filled shapes) remain largely unaffected for both ICP1 and PLE.

In this study, we used BREX and DarTG anti-phage defense systems as tools to probe the regulatory logic of PLE gene expression. We first established that, while both systems completely block the ICP1 helper phage’s genome replication, they exert distinct effects on the phage’s transcriptional program (Fig. 6). BREX imposes a profound arrest, restricting ICP1 gene expression to a small subset of immediate-early genes and preventing the accumulation of mid- and late-stage proteins. In contrast, DarTG is relatively permissive, allowing the phage transcriptional program to proceed almost entirely unperturbed. This culminates in the production of early, mid, and late proteins despite the block in DNA synthesis. The latter finding was somewhat surprising, given previous reports that plasmid-based expression of DarTG in *Escherichia coli* substantially reduced RNA synthesis following phage infection (7). This discrepancy may arise from key technical differences; while plasmid-based overexpression can cause non-physiological effects, our study examined the system in its native genetic context, potentially yielding more physiologically relevant insights. Alternatively, since neither ICP1 nor the previously evaluated coliphages encode their own RNA polymerase, the differing impacts of DarTG on RNA synthesis may reflect distinct mechanisms by which these phages hijack and modify the host transcriptional machinery. This highlights how defense phenotypes can inherently vary across different phage-host pairs.

The contrasting transcriptional landscapes imposed by these defense systems provided a unique opportunity to investigate PLE regulation, as neither background permits PLE replication. To untangle whether the resulting PLE transcriptional profiles were a consequence of this shared replication deficiency, we characterized the transcriptome of a replication-deficient (Δ*repA*) PLE mutant. Our results revealed that PLE still robustly expresses nearly its entire transcriptional program even without replicating, demonstrating that PLE gene expression and structural protein production are effectively uncoupled from its own replication. Hence, upon excision from the host chromosome to escape chromosomal degradation (26), a single episomal copy of PLE is sufficient to execute a robust gene expression program. Curiously, *orf7*, encoding a predicted MarR-like transcription factor, emerged as the only PLE gene significantly impacted by PLE’s replication status. Although the function of ORF7 remains unknown and it is dispensable for ICP1 inhibition (31), its unique dependence on replication suggests it may play a regulatory role in the late stages of infection, perhaps optimizing PLE horizontal transfer.

In the presence of BREX, we observed a transcriptional stall in PLE gene expression that closely mirrors the arrested state of the ICP1 helper phage developmental program. Previous studies indicate that BREX acts early during infection, primarily by blocking phage DNA replication (3), though the precise mechanism of phage restriction remains unclear. To our knowledge, no prior studies have investigated whether BREX impacts gene expression of the invading phage. Here, we found that in the presence of BREX, ICP1 initiates expression of immediate-early genes but fails to reach full early-, mid-, or late-stage expression. Although BREX could render incoming viral DNA transcriptionally inaccessible, such direct silencing alone does not explain the lack of PLE expression. BREX distinguishes self-DNA from unmodified foreign DNA through epigenetic methylation (4). The integrated PLE, being part of the host chromosome, is therefore expected to carry these native modifications. Since host transcription is generally unaffected by BREX-mediated restriction (Tables S1 and S2), and a subset of host genes is even upregulated, the observed stall in PLE expression most likely reflects a downstream consequence of ICP1 arrest, wherein the phage is unable to produce a necessary trans-acting factor(s). Indeed, this early window of infection appears to trigger modest transcriptomic induction of the PLE replication module; however, most of the remaining PLE modules remain transcriptionally silent in the BREX(+) background, and PLE proteins do not accumulate to detectable levels. This pattern suggests either that the cue(s) required to fully license robust transcription is produced only as the phage lifecycle progresses or that the initial low-level activation is insufficient to reach the expression threshold needed to activate additional operons.

A central question is how PLE senses the progression of its helper phage to initiate transcription. While PLE may directly respond to phage-encoded proteins, our data also support a model in which activation depends on modulation of host factors as the ICP1 lifecycle advances. The global host regulator H-NS is the primary repressor of PLE gene expression (21), and PLE transcriptional activation likely involves overcoming this repression. Operon-specific activation could indicate that multiple, distinct helper cues are produced sequentially; however, it is also consistent with a single anti-repressor model, in which an early expressed ICP1 factor relieves H-NS-mediated repression. In this model, different H-NS binding affinities at PLE promoters would create variable thresholds for derepression, potentially driving a staggered, temporal cascade of activation.

The regulatory logic governing PLE activation can be contextualized by existing paradigms of satellite induction. In the SaPI model, a single non-essential protein typically suffices to relieve repression and trigger the entire SaPI lifecycle (19, 20). Although to our knowledge, global assessments of satellite activation during helper phage infection have not been carried out outside the PLE-ICP1 system, evidence from another satellite shows that helper dependence can be more modular and distributed than the simple one anti-repressor/one repressor paradigm. Specifically, activation of the unrelated satellite P4 requires two distinct helper-encoded inputs for full induction by its cognate phage (32, 33). Independent gating of replication and morphogenesis operons in satellites may ensure that replication functions are rapidly available upon infection, whereas structural genes are expressed only after the helper phage reaches a stage compatible with satellite packaging and transfer. This multi-layered regulatory architecture is likely less vulnerable to phage escape via simple bypass of a single sensing mechanism. The observed evolutionary resilience of PLE, in which ICP1 appears unable to escape triggering PLE activation, could result from multiple distinct helper cues (as in P4), the required accumulation of a single, immutable phage protein essential to the phage’s lifecycle, or functional redundancy with secondary factors backing up a primary trigger. Determining whether PLE activation depends on a single master trigger or multiple phage-derived inputs will be an important direction for future mechanistic studies.

## MATERIALS AND METHODS

### Generation of appropriate controls for the experimental design

Two anti-phage defense systems that block the initiation of ICP1 genome replication in clinical isolates of *V. cholerae* were used in this study: BREX, encoded by *Vch*Ind5, an integrative and conjugative element (22), and DarTG, a toxin-antitoxin system encoded by a distinct phage defense element (23). To enable appropriate comparisons, we separately introduced the genetic elements encoding BREX and DarTG into the same parental *V. cholerae* strain harboring PLE1 (see “Conjugations and transductions,” below). To generate isogenic controls, we deleted hotspot five (encoding BREX) from *Vch*Ind5 (22), and designated it as BREX(−). As a permissive background for phage defense element encoding DarTG, we deleted *darT* (the toxin, shown to inhibit ICP1 genome replication in its native genetic context [23]) and also *old*, encoded by the same element but only shown to inhibit ICP1 when expressed from a plasmid (23, 34), and designated the resulting strain as DarTG(−). For all infections, we used a Δ*orbA* derivative of ICP1_2006_Dha_E ∆CRISPR-cas ∆*cas2-3*, as OrbA is the known inhibitor of the *Vch*Ind5 BREX system (22). The deletion of the ICP1 CRISPR-Cas system was necessary to prevent ICP1-mediated anti-PLE activity.

### Bacterial strains and growth conditions

Bacterial strains used in this study are listed in Table S14. All *V. cholerae* and *E. coli* strains were grown with aeration in a roller at 250 rpm at 37°C in lysogeny broth (LB). Where necessary, antibiotics were used at the following concentrations: 100 μg/mL streptomycin, 75 μg/mL kanamycin, 100 μg/mL spectinomycin, 32 μg/mL trimethoprim, 1.25 μg/mL chloramphenicol (*V. cholerae* in liquid), 2.5 μg/mL chloramphenicol (*V. cholerae* on plates), and 25 μg/mL chloramphenicol (*E. coli*). For induction of plasmids in *V. cholerae*, 1 mM isopropyl-β-d-thiogalactopyranoside (IPTG) and 1.5 mM theophylline were added when cultures reached an optical density (OD_600_) of 0.2 and were grown for additional 20 min at 37°C in the roller. For induction of *tcaP* expression in *E. coli* as a positive control for western blotting, 1 mM IPTG was added to cultures at an OD_600_ of 0.2 and cultures were grown for an additional 20 min at 37°C in the roller.

### Generation of mutant strains

Chromosomal deletions in *V. cholerae* were constructed by PCR amplifying 1 kb upstream and downstream homology arms, which then joined with a spectinomycin resistance cassette flanked by FRT (flippase recognition target) sites using splicing by overlap extension PCR. The stitched PCR product containing the arms of homology and the selective marker was then introduced into *V. cholerae* by natural transformation. To achieve natural competency, cells were grown in LB at 30°C until OD_600_ = 0.3. The cells were then pelleted and washed twice with 0.5× Instant Ocean. Then, cells were added to 80 mg chitin (Sigma) in 0.5× Instant Ocean and incubated for 24 h at 30°C. The transformants were plated on LB agar containing antibiotic. To remove the spectinomycin cassette, the inducible plasmid encoding the flippase was conjugated from *E. coli* S17 into *V. cholerae* and exconjugants were selected on streptomycin and chloramphenicol. Flippase expression was induced using 1 mM IPTG with 1.5 mM theophylline. Removal of the spectinomycin cassette and loss of the plasmid were verified by plating on LB agar plates supplemented with spectinomycin and chloramphenicol. Colonies that did not grow on spectinomycin or chloramphenicol plates were purified on LB agar supplemented with streptomycin, and the deletion was confirmed by PCR and Sanger sequencing.

### Conjugations and transductions

To obtain PLE(+) *Vch*Ind5(+) cells, *V. cholerae* E7946 PLE(+) was conjugated with *V. cholerae Vch*Ind5(+) donors (wild-type and the Δhotspot 5 BREX(−) derivative). *Vch*Ind5 donor *V. cholerae* strains were grown overnight with aeration in a roller at 250 rpm at 37°C in LB medium supplemented with trimethoprim, and PLE (+) recipient strains were grown with kanamycin. Five hundred microliters of overnight-grown donor and recipient cultures was mixed and pelleted at 5,000 × *g* for 3 min. Pellets were resuspended in 50 μL LB and pipetted onto a 0.8 μm filter disk on an LB agar plate. Conjugations were allowed to proceed for 6 h at 37°C. After the incubation, the filter disk was removed and added to a microcentrifuge tube with 1 mL LB. The microcentrifuge tube was vigorously vortexed to allow bacteria to dislodge from the filter into the liquid LB, and a dilution series was plated on trimethoprim and kanamycin to select for PLE(+) recipients that acquired *Vch*Ind5. Exconjugants were then purified further on trimethoprim and kanamycin plates two more times.

To obtain the PLE(+) DarTG(+) strain, PLE was transduced into the recipient DarTG(+) *V. cholerae* (23). Donor PLE(+) *V. cholerae* was grown with aeration in a roller at 250 rpm at 37°C in LB to OD_600_ = 0.3. Recipient DarTG(+) cells were grown overnight in the same conditions. Donor PLE(+) cells were infected with ICP1 at an MOI of 2.5, then the culture was washed twice to remove unbound phage and resuspended in LB with 10 mM MgCl_2_. After lysis, the culture was treated with chloroform, and the resulting lysate was centrifuged at 5,000 × *g* for 15 min at 4°C. The supernatant, which contains the PLE transducing particles, was added to the overnight recipient culture supplemented with 10 mM MgCl_2_ in a 1:1 ratio and incubated for 20 min with aeration at 250 rpm at 37°C. Transductants were plated on kanamycin and spectinomycin to select for recipients that acquired PLE. To obtain the otherwise isogenic PLE(+) DarTG(−) strain, *darT* and *old* were deleted in this background as described above.

### Quantification of genome replication by quantitative polymerase chain reaction (qPCR)

We used an established protocol to quantify phage genome replication as previously described (11). Briefly, *V. cholerae* strains were grown on a plate with an appropriate antibiotic overnight at 37°C. The next day, the strain was grown to an OD_600_ = 1 in liquid culture. Then, the liquid culture was back-diluted to 0.05 and grown to 0.3 and infected with ICP1 Δ*orbA* strain at a multiplicity of infection (MOI) of 0.1. Immediately after infection (*t* = 0), 100 μL of the samples was collected and boiled at 95°C for 10 min. The remaining culture was incubated at 37°C with aeration for 20 min (*t* = 20), 100 μL of the culture was collected, and boiled. Phage DNA from boiled samples was diluted 20-fold and measured by qPCR with primers zac68 (CTGAATCGCCCTACCCGTAC) and zac69 (GTGAACCAACCTTTGTCGCC) using iQ SYBR Green Supermix (Bio-Rad) and the CFX Connect Real-Time PCR Detection system (Bio-Rad). DNA replication was determined by quantifying the fold change in replication at *t* = 20 compared to genome copy at *t* = 0 using the *C*_*q*_ value relative to that of a standard curve of known concentrations of ICP1 genomic DNA. PLE replication followed the same protocol as above, with the following exceptions: *t* = 0 is collected immediately before infection, strains were infected with ICP1 at an MOI = 2.5, samples were diluted 500-fold before being used as templates for qPCR reactions, and primers zac14 (AGGGTTTGAGTGCGATTACG) and zac15 (TGAGGTTTTACCACCTTTTGC) specific for the PLE genome were used. For both phage and PLE genome replication, all conditions were tested in biological triplicate, and each reported data point is the mean of three technical replicates.

### Western blot analysis

*V. cholerae* strains were grown on a plate with an appropriate antibiotic overnight at 37°C. The next day, the strain was grown to an OD_600_ = 1 in liquid culture. Next, the liquid culture was back-diluted to an OD_600_ = 0.05 and grown to an OD_600_ = 0.3. Then, a 1 mL aliquot of the culture was spun down at 5,000 × *g* for 3 min. The remaining culture was infected with ICP1 at an MOI of 2.5. For each time point post-infection indicated in the figure, a 1 mL aliquot of culture was collected and mixed with ice-cold methanol and centrifuged at 5,000 × *g* for 3 min. Cell pellets were resuspended in Laemmli buffer supplemented with β-mercaptoethanol (Bio-Rad). Samples were boiled for 10 min at 99°C before loading 10 μL onto the Mini-Protean TGX Stain Free Precast PAGE gels (Bio-Rad). For quantifying the relative abundance of the protein of interest in different conditions, total protein was quantified by activating stain-free gels for 2 min on the UV/Stain-Free tray and imaged on Gel Doc EX Imager (Bio-Rad), and protein band intensities were measured using Fiji software (Version 2.16.0). The gel was then transferred to a mini-size PVDF membrane (Bio-Rad) via the Trans-Blot Turbo system. Proteins were detected via custom primary peptide antibodies generated in rabbits against PLE-encoded TcaP, RepA, or ICP1-encoded DNAP, PexA, and Gp122 (GenScript). Primary antibodies were used at the following dilutions: RepA and Gp122 (1:1,500), PexA (1:15,000), DNAP (1:1,000), and TcaP (1:600). Secondary detection was performed using goat anti-rabbit IgG horseradish peroxidase antibody (Bio-Rad, diluted 1:10,000). The membrane was then developed with Clarity Western ECL substrate (Bio-Rad) and imaged on a ChemiDoc XRS imaging system (Bio-Rad). The intensity of the band corresponding to the protein of interest (verified by size and appropriate controls as indicated on the blots) was normalized to the total protein loaded to each well.

### RNA isolation for RNA-seq

Three biological replicates of RNA isolations were conducted for each background. *V. cholerae* strains were grown on a plate with an appropriate antibiotic overnight at 37°C. The next day, the strain was grown to an OD_600_ = 1 in liquid culture. Next, the liquid culture was back-diluted to an OD_600_ = 0.05 and grown to an OD_600_ = 0.3. A 4 mL sample was removed before infecting with ICP1 at an MOI of 2.5, and 4 mL sample was collected at 8 and 16 mpi; all samples were immediately mixed with an equal volume of ice-cold methanol and pelleted at 5,000 × *g* for 5 min at 4°C. The supernatant was removed, and the pellets were washed with ice-cold 1× phosphate-buffered saline (pH 6.2). The mixture was pelleted at 5,000 × *g* for 5 min at 4°C, the supernatant was removed, and pellets were resuspended in 200 μL of TRIzol Reagent (Invitrogen). The samples were stored at −80°C. To extract the RNA, the samples were thawed at room temperature (RT) for 5 min and 40 μL chloroform was added to each sample. Samples were vigorously vortexed and incubated at RT for another 10 min. Then, to separate the phases, samples were centrifuged at 12,000 × *g* for 10 min at 4°C. The top aqueous layer was mixed thoroughly with 110 μL of isopropanol and 11 μL of 3 M sodium acetate (pH 7.4) and incubated at RT for 10 min. Samples were then centrifuged at 12,000 × *g* for 15 min at 4°C. The supernatant was discarded, and the pellet containing the RNA was washed twice with 500 μL 75% ethanol. To evaporate the remaining ethanol, the pellets were incubated at 65°C for at least 2 min. Finally, RNA pellets were resuspended in 20 μL of RNAse-free water.

### RNA-seq analysis

Isolated RNA samples were sequenced by the SeqCenter (Pittsburgh, PA, USA) with a sequencing depth of 12 million reads. The paired-end FASTQ files were mapped to the reference genomes using bowtie2 (35). Reference genomes were generated *in silico* as required to generate the appropriate *V. cholerae* chromosomes 1 (CP024162.1) and 2 (CP024163.1) and introducing the integrative conjugative element SXT *Vch*Ind5 (GQ463142) or phage defense element encoding DarTG (23). The resulting mapped alignment files were converted to BAM format, sorted, and indexed using samtools (36). Transcript abundance was estimated using StringTie (37). Transcript count matrices were generated using the prepDE.py script provided with StringTie. The matrices were imported into RStudio for downstream differential expression analysis using DESeq2 (38). Variance-stabilizing transformed expression values were used for PCA to assess data quality and sample clustering. One biological replicate for BREX(+) cells in the uninfected condition clustered aberrantly in the PCA plot and was excluded as an outlier for the comparison between BREX(+) infected and BREX(+) uninfected samples. Therefore, differential expression analysis was performed using two biological replicates for the BREX(+) uninfected condition, while three biological replicates were used for all other conditions and comparisons. Results were illustrated using volcano plots generated using GraphPad [version 10.5.0 (673)].

Sorted and indexed BAM files were used to generate strand-specific RNA-seq coverage tracks. Normalization factors were calculated using multiBamSummary, and strand-specific coverage tracks were generated using bamCoverage from deepTools (39). Coverage tracks were exported as BedGraph files and merged across biological replicates using unionbedg from BEDTools (40) to compute mean coverage for each condition. The resulting normalized strand-specific coverage tracks were visualized across ICP1 and PLE genomes together with gene annotations from the GTF file.

The proportion of transcript abundance for each element in the sample (percent reads) was calculated by normalizing raw integer counts to the total library size. Reads mapping to PLE, ICP1, and the host genome were aggregated by element and divided by the total library reads and expressed as a percentage of the total reads per sample. Results were illustrated using comparative bar graphs using GraphPad [version 10.5.0 (673)].

## Supplementary Material

Reviewer comments

## Data Availability

Sequence data for samples used in this work can be found in the Sequence Read Archive under BioProject accession PRJNA1439424.

## SUPPLEMENTAL MATERIAL

Supplemental material msystems.00428-26-s0001.pdf Fig. S1-S7 and Table S14.

Supplemental tables msystems.00428-26-s0002.xlsx Tables S1-S13.

OPEN PEER REVIEW reviewer-comments.pdf An accounting of the reviewer comments and feedback.

ASM does not own the copyrights to Supplemental Material that may be linked to, or accessed through, an article. The authors have granted ASM a non-exclusive, world-wide license to publish the Supplemental Material files. Please contact the corresponding author directly for reuse.
