# Supplementary material for: Repurposing anti-phage defenses to differentially arrest the viral lifecycle reveals the regulatory logic of a parasitic satellite
Source: mSystems. 2026 Sep 2;11(9):e00428-26. doi: 10.1128/msystems.00428-26 (PMC13595925; doi:10.1128/msystems.00428-26)
Supplement: Supplementary file 2 [file reviewer-comments.pdf]

ASM | mSystems

Repurposing anti-phage defenses to differentially arrest the viral lifecycle reveals the regulatory logic of a parasitic satellite

Sevgi Bagdatli and Kimberley Seed

Corresponding Author(s): Kimberley Seed, University of California Berkeley

**Review Timeline:**		
	Submission Date:	April 6, 2026
	Editorial Decision:	May 3, 2026
	Revision Received:	July 29, 2026
	Accepted:	August 7, 2026

Editor: Mark Mandel

Reviewer(s): The reviewers have opted to remain anonymous.

Transaction Report:

(Note: With the exception of the correction of typographical or spelling errors that could be a source of ambiguity, letters and reports are not edited. The original formatting of letters and referee reports may not be reflected in this compilation.)

DOI: https://doi.org/10.1128/msystems.00428-26

Re: mSystems00428-26 (**Repurposing anti-phage defenses to differentially arrest the viral lifecycle reveals the regulatory logic of a parasitic satellite**)

Dear Dr. Kimberley D. Seed:

Thank you for the privilege of reviewing your work. Below you will find my comments, instructions from the mSystems editorial office, and the reviewer comments.

Both reviewers had favorable feedback about the work overall. At the same time, they each pointed out areas where interpretation of the findings in the manuscript was not fully supported (or sufficiently clear, in the case of Review 2, #3) from the data presented. In your revised manuscript, be sure that the points from both reviewers are fully addressed. While you may opt to include new data to address these points, I believe that the comments can be resolved through alterations in the manuscript without the need for new experiments.

Please return the manuscript within 60 days; if you cannot complete the modification within this time period, please contact me. If you do not wish to modify the manuscript and prefer to submit it to another journal, notify me immediately so that the manuscript may be formally withdrawn from consideration by mSystems.

Revision Guidelines
To submit your modified manuscript, log into the submission site at https://msystems.msubmit.net/cgi-bin/main.plex. Go to Author Tasks and click the appropriate manuscript title to begin. The information you entered when you first submitted the paper will be displayed; update this as necessary. Note the following requirements:

• Upload point-by-point responses to the issues raised by the reviewers in a file named "Response to Reviewers," NOT in your cover letter.
• Upload a compare copy of the manuscript (without figures) as a "Marked-Up Manuscript" file.
• Upload a clean .DOC/.DOCX version of the revised manuscript and remove the previous version.
• Each figure must be uploaded as a separate, editable, high-resolution file (TIFF or EPS preferred), and any multipanel figures must be assembled into one file.
• Any supplemental material intended for posting by ASM should be uploaded with their legends separate from the main manuscript. You can combine all supplemental material into one file (preferred) or split it into a maximum of 10 files with all associated legends included.

For complete guidelines on revision requirements, see our Submission and Review Process webpage. Submission of a paper that does not conform to guidelines may delay acceptance of your manuscript.

Data availability: ASM policy requires that data be available to the public upon online posting of the article, so please verify all links to sequence records, if present, and make sure that each number retrieves the full record of the data. If a new accession number is not linked or a link is broken, provide mSystems production staff with the correct URL for the record. If the accession numbers for new data are not publicly accessible before the expected online posting of the article, publication may be delayed; please contact production staff (mSystems@asmusa.org) immediately with the expected release date.

Publication Fees: For information on publication fees and which article types are subject to charges, visit our website. If your manuscript is accepted for publication and any fees apply, you will be contacted separately about payment during the production process; please follow the instructions in that e-mail. Arrangements for payment must be made before your article is published.

ASM Membership: Corresponding authors may join or renew ASM membership to obtain discounts on publication fees. Need to upgrade your membership level? Please contact Customer Service at Service@asmusa.org.

The ASM Journals program strives for constant improvement in our submission and publication process. Please tell us how we can improve your experience by taking this quick Author Survey.

Thank you for submitting your paper to mSystems.

Sincerely,
Mark Mandel
Editor
mSystems

Reviewer #1 (Comments for the Author):

This manuscript by Bagdatli and Seed is an interesting approach using two phage defense systems in Vibrio cholerae, BREX and DarTG, to probe the transcriptional activation program of the phage ICP-1 and its satellite, parasitic phage PLE. Although PLE is known to be activated upon ICP-1 infection, it is unclear what aspect of ICP-1 infection triggers the cascade of PLE gene expression. Both defense systems block replication of the two phages. The authors find that BREX significantly reduces expression of virtually all ICP-1 genes (except for 11 early genes) and reduces expression of all PLE genes while DarTG does not significantly inhibit transcription of either phage. This suggests an interesting finding that the transcriptional program of each phage is not linked to phage replication, which the authors subsequently confirm for PLE using a repA mutant that cannot replicate. I agree with this conclusion. My issue with the manuscript is the author's proposal that the results observed demonstrate that PLE activation differs from the canonical model (developed in the SaPI satellite phages in Staphylococcus aureus) whereby one phage anti-repressor protein activates the parasitic phage. As argued (somewhat redundantly-sorry) in points 1, 7, and 8, I think the authors are overinterpreting that their data shows PLE is activated by "multiple distinct cues" and their results could also be observed in a SaPI-type of system. I think the main difference between my view and the authors is they conclude PLE early genes are activated in a BREX+ cell whereas my interpretation of their results is the minimal induction of the early PLE in the BREX+ cell is insignificant as it is order of magnitude less than a BREX- cell (point 5). Outside of this discrepancy of interpretation, the data are very clear and the manuscript is well written and has several interesting findings including the activity of BREX and DarTG in a native host/phage/satellite system that will be of significant interest to the field.

1. Lines 360-362-I don't understand the logic for stating that the data show implies that PLE activation requires multiple distinct cues. I think it is just as possible that the PLE has a specific transcriptional program that is initiated but blocked by BREX such that it never moves past a weak induction of early genes. In other words, the early genes of PLE never reach the expression threshold necessary to activate middle and late genes. This model is supported by Fig. 4E where the early genes proteins are undetectable by Western blot even though they are minimally induced.
2. Line 371-I think this sentence proposing that the lack of visible proteins by Western blot "strongly suggests" post-transcriptional regulation mechanisms is too speculative given the relatively poor induction of these genes in the presence of BREX (greater than 500-fold less induction than the BREX- mutant).
3. Fig. 4 and 5-it might be worth adding forward and reverse strands to the RNA-Seq data as I was confused for a bit about what was represented in the top and bottom sections of the graph before checking the legend.
4. Line 409-I would change "may" to "likely". I am surprised the PLE transcripts are not lower than 4% given that there are ~100 less genomes. This suggests to me that many of the genomes are not actually producing transcripts (or perhaps some factor needed for transcription is limited as PLE genomes increase).
5. Lines 459-461-I think the authors overstate the induction of early PLE genes here in the presence of BREX. Yes, there was a measurable induction, but as stated above it was orders of magnitude less than a BREX- cell and thus I would argue the early genes were not significantly induced in the presence of BREX.
6. Line 466-What phages were used in Ref 31 demonstrating that DarTG reduced RNA synthesis? Are they related to ICP-1? This result seems quite dependent on specific phage encoded RNA polymerases.
7. Line 506-I am not convinced of the model of "progressive licensing of PLE during ICP1 infection". As BREX inhibits transcription of ICP1, how can the authors rule out a model whereby an early essential gene of ICP1 is necessary for PLE induction, but that protein is not made in the presence of BREX (or it is made at very low levels) and thus PLE is not induced? Indeed, in lines 203-204 the authors state that at 8 minutes post infection only 11/227 ICP1 genes were not differentially expressed. If the trigger for PLE induction is not one of these 11 genes, then I would expect similar results for PLE induction that the authors observed. I would guess the authors would point to induction of the early PLE genes in the presence of BREX as evidence of its activation, but as I argue in point 5 this induction seems insignificant compared to the normal PLE activation.
8. To continue with the above point, what do the authors envision they would see with these systems and the SaP1 model using their system? If BREX (or some similar defense) significantly curtails transcription induction of the phage anti-repressor, but maybe a very small amount is produced before inhibition (similar to their observations for PexA in this manuscript where they saw activity but no observable protein-lines 274-275), then early gene transcription of the SaP1 phage might show very weak induction but not be triggered for full activation similar to their observations for PLE here. How do their results rule out such a model? Note I am not referring to phage excision here, but the transcriptional progression of phage gene expression.

Reviewer #2 (Comments for the Author):

In Bagdatli & Seed (2026), the authors investigate the effect of two DNA replication blocking antiphage defense systems on the ICP1 phage transcription, as well as on transcription of the PICI element PLE1. They propose using this transcriptional analysis to better understand PLE1 activation - which, in the absence of escape mutants and a single known "trigger" could be due to more complex multi-layered signals - they induce two antiphage defense systems that inhibit DNA replication, BREX and DarTG. The two defense systems act via different mechanisms to block DNA: the precise mechanism of BREX is unknown while DarT ADP-ribosylates DNA. While both fully block DNA replication, they have different effects on ICP1 phage: BREX inhibits nearly all phage and severely limits PLE1 gene expression, while more mild changes in transcription occur with DarTG1. They first show that PLE expression is not dependent on replication by examining a ΔrepA mutant, then compare that to gene expression in the BREX treated cells. Expression is normal in ΔrepA while in BREX expression is severely limited, and somewhat limited in DarTG cells. They interpret this to mean that an ICP1 factor is required for PLE transcription, but another possibility is that BREX prevents phage transcription directly, just as it limits ICP1 transcription. The authors conclude that PLE regulation and activation

is intrinsically tied to the transcriptional lifecycle of its helper phage.

The RNA-seq is a valuable dataset to publish and seems well executed, and the authors have performed some additional supporting experiments (e.g. examining translation vs transcription). This is not a complete story, but the decoupling of replication from transcription is a worthwhile result to report as it appears to differ from T4 phage and some eukaryotic viruses, and is one more piece in the PLE1 activation puzzle. However, the authors have over-interpreted these results and either need to provide more evidence to support their conclusions, or modify the conclusions as reported here.

Major

1. My major concern is that while this manuscript demonstrates that PLE and ICP1 expression are uncoupled from replication and that DarTG and BREX inhibit expression of PLE and ICP1 genes on different time scales, the conclusion "the sequential activation of PLE operons suggests that each operon is derepressed as distinct milestones of the ICP1 developmental program are achieved" is unsupported. The effects of BREX and DarTG1 on PLE mirror what is seen for ICP1 in both DNA replication and transcription. The simplest explanation is that DarT ADP-ribosylation of the genome is responsible for reducing PLE transcription, just as it mildly reduces ICP1 transcription. Thus, the reduced levels of transcription are not evidence of a multi step activation/licensing process, but simply the result of inefficient transcription when DNA is ADP-ribosylated. The conclusions reached from these data must be altered to reflect this strong possibility.

2. One complicating factor is that neither of the mechanisms of these defense systems are thoroughly understood, thus using them as molecular tools is challenging to interpret. While it is known that DarT poisons DNA via ADP-ribosylation, as the authors point out, a previous study reported that transcription is blocked in the two phages examined. Clearly for ICP1, transcription is not fully blocked by DNA ADP-ribosylation. For BREX, the mechanism of the DNA replication block is not known. Previously studies report that the DNA is not degraded, but perhaps it is inactivated in such a way as to make DNA completely inaccessible for transcription? This would be consistent with the fact that no transcription is seen for ICP1, and only very low levels for PLE.

3. A schematic summarizing the different impacts on activation (DNA replication, transcription, translation) of ICP1 and PLE1 in response to DarTG and BREX would be very helpful to the reader. This was somewhat confusing to parse as currently presented. Something that summarizes the below information, if I understand it correctly:
BREX-> fully blocks ICP1 replication + transcription -> permissive to PLE1 circularization, fully blocks PLE1 replication, partially reduces transcription, no translation detected of 2 proteins examined
DarTG: fully blocks ICP1 replication, partially transcription -> permissive to PLE1 circularization, fully blocks PLE1 replication, partially reduces transcription, robust of 2 proteins examined.

Response to Reviewers

We thank the editor and reviewers for their insightful feedback and encouraging assessment of our manuscript. In this revised version, we have carefully addressed all reviewer concerns, particularly by clarifying our interpretations and toning down specific conclusions where noted. Below, we provide detailed, point-by-point responses to each comment and outline the corresponding revisions made to the text.

Reviewer #1 (Comments for the Author):

This manuscript by Bagdatli and Seed is an interesting approach using two phage defense systems in Vibrio cholerae, BREX and DarTG, to probe the transcriptional activation program of the phage ICP-1 and its satellite, parasitic phage PLE. Although PLE is known to be activated upon ICP-1 infection, it is unclear what aspect of ICP-1 infection triggers the cascade of PLE gene expression. Both defense systems block replication of the two phages. The authors find that BREX significantly reduces expression of virtually all ICP-1 genes (except for 11 early genes) and reduces expression of all PLE genes while DarTG does not significantly inhibit transcription of either phage. This suggests an interesting finding that the transcriptional program of each phage is not linked to phage replication, which the authors subsequently confirm for PLE using a repA mutant that cannot replicate. I agree with this conclusion. My issue with the manuscript is the author's proposal that the results observed demonstrate that PLE activation differs from the canonical model (developed in the SaPI satellite phages in Staphylococcus aureus) whereby one phage anti-repressor protein activates the parasitic phage. As argued (somewhat redundantly-sorry) in points 1, 7, and 8, I think the authors are overinterpreting that their data shows PLE is activated by "multiple distinct cues" and their results could also be observed in a SaPI-type of system. I think the main difference between my view and the authors is they conclude PLE early genes are activated in a BREX+ cell whereas my interpretation of their results is the minimal induction of the early PLE in the BREX+ cell is insignificant as it is order of magnitude less than a BREX- cell (point 5). Outside of this discrepancy of interpretation, the data are very clear and the manuscript is well written and has several interesting findings including the activity of BREX and DarTG in a native host/phage/satellite system that will be of significant interest to the field.

1. Lines 360-362-I don't understand the logic for stating that the data show implies that PLE activation requires multiple distinct cues. I think it is just as possible that the PLE has a specific transcriptional program that is initiated but blocked by BREX such that it never moves past a weak induction of early genes. In other words, the early genes of PLE never reach the expression threshold necessary to activate middle and late genes. This model is supported by Fig. 4E where the early genes proteins are undetectable by Western blot even though they are minimally induced.

Response: We thank the reviewer for bringing up this point. We agree that our data do not definitively rule out an threshold-dependent model. To provide a more complete and nuanced picture, we have updated the manuscript to highlight our 8 mpi transcriptomic

data. At 8 mpi, *repA* transcript levels show a statistically significant 22-fold induction in the BREX(+) background compared to a ~330-fold induction in the BREX(−) control. While this indicates a genuine, discernible upward shift early in infection (within a single order of magnitude of the control), this developmental stall is heavily exacerbated by 16 mpi and ultimately fails to yield detectable protein.

Because current data cannot definitively distinguish whether this phenotype is due to a lack of distinct downstream viral cues or a failure to cross a critical expression threshold, we have removed the definitive claim regarding multiple distinct cues. We have revised the text to present both interpretations as equally plausible models:

"*This selective, low-level activation could point to a multi-cue licensing model, in which immediate-early phage input(s) trigger the PLE early operon, but downstream modules remain silent because the arrested phage lifecycle fails to reach the specific milestones required to activate them. Alternatively, the data are consistent with a model in which trace levels of a specific phage-derived triggering factor do not reach the threshold necessary for complete and robust transcriptional activation of the satellite.*"

2. Line 371-I think this sentence proposing that the lack of visible proteins by Western blot "strongly suggests" post-transcriptional regulation mechanisms is too speculative given the relatively poor induction of these genes in the presence of BREX (greater than 500-fold less induction than the BREX- mutant).

Response: We agree that the phrase "strongly suggests" was too speculative, however, because post-transcriptional regulation remains an explanation for our observations, we have revised the manuscript to present this as a possibility rather than a strong conclusion "..*this result suggests that BREX may impose an additional block on gene expression post-transcriptionally".*

3. Fig. 4 and 5-it might be worth adding forward and reverse strands to the RNA-Seq data as I was confused for a bit about what was represented in the top and bottom sections of the graph before checking the legend.

Response: We thank the reviewer for this recommendation. We have updated Figures 4 and 5 to explicitly label the forward (+) and reverse (-) strands directly on the y-axes for clarity.

4. Line 409-I would change "may" to "likely". I am surprised the PLE transcripts are not lower than 4% given that there are ~100 less genomes. This suggests to me that many of the genomes are not actually producing transcripts (or perhaps some factor needed for transcription is limited as PLE genomes increase).

Response: We have changed "may" to "likely" in the revised text.

5. Lines 459-461-I think the authors overstate the induction of early PLE genes here in the presence of BREX. Yes, there was a measurable induction, but as stated above it was

orders of magnitude less than a BREX- cell and thus I would argue the early genes were not significantly induced in the presence of BREX.

Response: We believe the reviewer is referring to the paragraph beginning on line 349 of the original draft "However, differential expression analysis comparing infected to uninfected BREX(+) cells revealed that a subset of genes, comprised of the early replication operon (*orf8* to *orf14)* and *nixI-tcaP*, is upregulated during infection…" As noted in our response to Point 1, our differential expression analysis demonstrates that the early PLE operon is statistically significantly upregulated during infection in the BREX(+) background. To address the reviewer's concern regarding the magnitude of this induction, we have revised the text to present the 8 mpi transcriptomic data explicitly. At this early time point, *repA* transcript levels increase 22-fold in BREX(+) cells compared to a ~330-fold increase in the BREX(−) control. This demonstrates that the initial activation of the early operon is within a single order of magnitude of the permissive control, rather than multiple orders of magnitude.

However, we entirely agree with the reviewer that it is unclear if this statistically significant upregulation translates to a biologically meaningful event. Because the transcripts fail to accumulate further at 16 mpi and do not yield detectable protein using our custom antibody, we have toned down our conclusions in this section and throughout the manuscript. To ensure this nuanced interpretation is carried throughout the entire manuscript, we have also updated the Discussion. We note that in the original draft, we explicitly characterized the transcriptomic response in the presence of BREX as a 'modest transcriptomic induction of the PLE replication module (Line 492 of the original manuscript)' to reflect its restricted nature. To fully accommodate the reviewer's feedback, we have modified this section to explicitly include the expression threshold model alongside the progressive licensing model:

"Indeed, this early window of infection appears to trigger modest transcriptomic induction of the PLE replication module; however, most of the remaining PLE modules remain transcriptionally silent in the BREX(+) background, and PLE proteins do not accumulate to detectable levels. This pattern suggests that the cue(s) required to fully license robust transcription are either produced only as the phage lifecycle progresses or that the initial low-level activation is insufficient to reach the expression threshold needed to activate additional operons."

Likewise, we have also edited the abstract to remove the claim about the progressive licensing strategy.

6. Line 466-What phages were used in Ref 31 demonstrating that DarTG reduced RNA synthesis? Are they related to ICP-1? This result seems quite dependent on specific phage encoded RNA polymerases.

Response: The reviewer raises an excellent point regarding the potential dependence of this phenotype on transcription machinery. The referenced study evaluated DarTG defense in *E. coli* using the coliphages RB69 and T5, which are not related to the *V.*

cholerae phage ICP1. Interestingly, neither RB69, T5, nor ICP1 encodes its own RNA polymerases; rather, they all rely on hijacking the host RNA polymerase. The discrepancy in the observed transcriptional block observed in the *E.coli* study and the landscape we observed for ICP1 may stem from differences in how these distinct phages modify the host polymerase. We agree that this is an important distinction to make clear for the reader and have updated the Discussion text to incorporate this explanation: "*Alternatively, since neither ICP1 nor the previously evaluated coliphages encode their own RNA polymerase, the differing impacts of DarTG on RNA synthesis may reflect distinct mechanisms by which these phages hijack and modify the host transcriptional machinery. This highlights how defense phenotypes can inherently vary across different phage-host pairs*."

7. Line 506-I am not convinced of the model of "progressive licensing of PLE during ICP1 infection". As BREX inhibits transcription of ICP1, how can the authors rule out a model whereby an early essential gene of ICP1 is necessary for PLE induction, but that protein is not made in the presence of BREX (or it is made at very low levels) and thus PLE is not induced?...

Response: We agree with this assessment. As noted in our responses to Points 1 and 5, we cannot formally rule out a model in which a single essential early ICP1 protein acts as the trigger but fails to reach the required concentration threshold due to BREX-mediated repression of the ICP1 transcriptional program. We have revised the manuscript to present this threshold-based mechanism as a strong alternative to the multiple-cue model.

8. To continue with the above point, what do the authors envision they would see with these systems and the SaP1 model using their system? If BREX (or some similar defense) significantly curtails transcription induction of the phage anti-repressor, but maybe a very small amount is produced before inhibition... How do their results rule out such a model?

Response: We appreciate the reviewer's insight. If a SaPI-type system were subjected to a BREX-mediated transcriptional block, a single phage-encoded anti-repressor failing to reach functional levels for full activation might be expected to cause either a complete block or uniform, low-level activation across the satellite. In our data, however, we see differential operon activation, where the early/replication operon is transcribed while downstream morphogenesis operons remain stalled. While operon-specific activation could suggest multiple distinct helper cues, it is also consistent with a single-trigger model if individual PLE promoters possess distinct affinity thresholds for a single helper factor, or if additional downstream controls are at play. As a result, we have shifted the narrative to emphasize that PLE transcription is tied to the global transcriptional progression of the phage and included the potential requirement to overcome a critical expression threshold for complete activation.

Reviewer #2 (Comments for the Author):

In Bagdatli & Seed (2026), the authors investigate the effect of two DNA replication blocking antiphage defense systems on the ICP1 phage transcription, as well as on transcription of the PICI element PLE1 They propose using this transcriptional analysis to better understand PLE1 activation - which, in the absence of escape mutants and a single

known "trigger" could be due to more complex multi-layered signals - they induce two antiphage defense systems that inhibit DNA replication, BREX and DarTG. The two defense systems act via different mechanisms to block DNA: the precise mechanism of BREX is unknown while DarT ADP-ribosylates DNA. While both fully block DNA replication, they have different effects on ICP1 phage: BREX inhibits nearly all phage and severely limits PLE1 gene expression, while more mild changes in transcription occur with DarTG1. They first show that PLE expression is not dependent on replication by examining a ΔrepA mutant, then compare that to gene expression in the BREX treated cells. Expression is normal in ΔrepA while in BREX expression is severely limited, and somewhat limited in DarTG cells. They interpret this to mean that an ICP1 factor is required for PLE transcription, but another possibility is that BREX prevents phage transcription directly, just as it limits ICP1 transcription. The authors conclude that PLE regulation and activation is intrinsically tied to the transcriptional lifecycle of its helper phage.

The RNA-seq is a valuable dataset to publish and seems well executed, and the authors have performed some additional supporting experiments (e.g. examining translation vs transcription). This is not a complete story, but the decoupling of replication from transcription is a worthwhile result to report as it appears to differ from T4 phage and some eukaryotic viruses, and is one more piece in the PLE1 activation puzzle. However, the authors have over-interpreted these results and either need to provide more evidence to support their conclusions, or modify the conclusions as reported here.

Major

1. My major concern is that while this manuscript demonstrates that PLE and ICP1 expression are uncoupled from replication and that DarTG and BREX inhibit expression of PLE and ICP1 genes on different time scales, the conclusion "the sequential activation of PLE operons suggests that each operon is derepressed as distinct milestones of the ICP1 developmental program are achieved" is unsupported. The effects of BREX and DarTG1 on PLE mirror what is seen for ICP1 in both DNA replication and transcription. The simplest explanation is that DarT ADP-ribosylation of the genome is responsible for reducing PLE transcription, just as it mildly reduces ICP1 transcription. Thus, the reduced levels of transcription are not evidence of a multi step activation/licensing process, but simply the result of inefficient transcription when DNA is ADP-ribosylated. The conclusions reached from these data must be altered to reflect this strong possibility.

Response: We appreciate the reviewer's perspective; however, our detailed operon-specific data strongly argue against a model in which ADP-ribosylation causes generic or non-specific "inefficient transcription."

If ADP-ribosylation of the template DNA broadly impaired transcriptional efficiency, we would expect uniform dampening across the entire satellite. Instead, we observe distinct, operon-specific transcriptional dynamics during DarTG defense (lines 411-423 of the original submission):

- At 8 mpi, early PLE gene expression is completely unaffected by DarTG defense, with all genes showing comparable upregulation in both DarTG(+) and DarTG(-) conditions.
- At 16 mpi, genes in the replication module (*orf8-orf13*, e.g., *repA* upregulated ~1,500-fold in DarTG(+) vs ~4,200-fold in control) and the packaging inhibition module (*gpsAB-capR*) still achieve robust, high-magnitude expression.
- In contrast, other genes show a selective and disproportionate defect at 16 mpi (e.g., the tail gene *tmp* increases ~230-fold in DarTG(+) compared to ~2,300-fold in control; *orf7* increases ~8-fold in DarTG(+) compared to ~640-fold in control).

The fact that replication and packaging-inhibition modules are transcribed at high levels while others are selectively stalled demonstrates that ADP-ribosylation does not simply interfere with transcription across the board. Rather, this operon-specific gating indicates that full expression of late PLE genes depends on the developmental progression of the helper phage and/or satellite lifecycle. We have clarified this point by adding this explanation to the revised manuscript (Lines 350-355).

Nevertheless, we acknowledge the reviewer's broader concern regarding how we framed these findings. We agree that our data should not be presented as definitive proof of a strict multi-cue licensing model. In the revised manuscript (including the Abstract, Results, and Discussion), we have softened our conclusions. We have shifted our focus away from claiming a multi-step licensing process and instead highlight the core discovery supported by our data: that late-stage viral and satellite gene expression can be decoupled from genome replication.

2. One complicating factor is that neither of the mechanisms of these defense systems are thoroughly understood, thus using them as molecular tools is challenging to interpret. While it is known that DarT poisons DNA via ADP-ribosylation. While it is known that DarT poisons DNA via ADP-ribosylation, as the authors point out, a previous study reported that transcription is blocked in the two phages examined. Clearly for ICP1, transcription is not fully blocked by DNA ADP-ribosylation. For BREX, the mechanism of the DNA replication block is not known. Previously studies report that the DNA is not degraded, but perhaps it is inactivated in such a way as to make DNA completely inaccessible for transcription? This would be consistent with the fact that no transcription is seen for ICP1, and only very low levels for PLE.

Response: We appreciate the reviewer raising this thoughtful mechanistic point. We fully agree that because the precise molecular mechanisms of BREX (and to a lesser extent DarTG) remain active areas of study, using them as molecular tools requires careful interpretation. Specifically, the reviewer posits that BREX might directly render phage DNA inaccessible to the transcription machinery, and asks whether a similar direct mechanism could explain the loss of PLE transcription without requiring an ICP1 factor. While we agree that rendering incoming viral DNA transcriptionally inaccessible could explain how

BREX silences ICP1, we respectfully disagree that this mechanism could account for the loss of PLE transcription, for the following reasons:

1. BREX operates as an epigenetic defense system that distinguishes host "self" DNA from invading "non-self" DNA via methylation. The PLE is integrated in the *V. cholerae* chromosome and is maintained as "self" DNA (which does not replicate upon infection in BREX(+) cells).

2. During BREX-mediated phage restriction, host transcription is maintained, and a subset of host bacterial genes are significantly upregulated (Table S1 and S2). This indicates that BREX does not cause widespread transcriptional silencing across the bacterial chromosome.

3. Because the chromosomal PLE locus is epigenetically marked as "self" DNA, any direct mechanism that BREX deploys to render incoming, unmethylated ICP1 DNA transcriptionally inaccessible would not target the host chromosome.

Consequently, the failure of PLE to undergo transcriptional activation during BREX defense is far more consistently explained as a downstream consequence: BREX restricts ICP1 at an immediate-early stage, preventing the phage from producing the trans-acting viral factor(s) or cues necessary to trigger PLE transcriptional activation.

We acknowledge that we did not explicitly lay out this logic in our original submission, which was an oversight on our part. In the revised manuscript, we have updated the Discussion (Lines 409-417) to explicitly walk through this rationale, clarifying why BREX restriction of ICP1—rather than direct action on the PLE genome—underlies the lack of PLE activation.

3. A schematic summarizing the different impacts on activation (DNA replication, transcription, translation) of ICP1 and PLE1 in response to DarTG and BREX would be very helpful to the reader. This was somewhat confusing to parse as currently presented. Something that summarizes the below information, if I understand it correctly: BREX-> fully blocks ICP1 replication + transcription -> permissive to PLE1 circularization, fully blocks PLE1 replication, partially reduces transcription, no translation detected of 2 proteins examined. DarTG: fully blocks ICP1 replication, partially transcription -> permissive to PLE1 circularization, fully blocks PLE1 replication, partially reduces transcription, robust of 2 proteins examined.

Response: We thank the reviewer for this recommendation. We have added a new summary schematic (Figure 6) that synthesizes the impacts of BREX and DarTG on ICP1 and PLE replication, transcription, and translation, incorporating the exact parameters outlined here. We believe this significantly improves the clarity of the manuscript.

Re: mSystems00428-26R1 (**Repurposing anti-phage defenses to differentially arrest the viral lifecycle reveals the regulatory logic of a parasitic satellite**)

Dear Dr. Kimberley D. Seed:

Your manuscript has been accepted, and I am forwarding it to the ASM production staff for publication. Your paper will first be checked to make sure all elements meet the technical requirements. ASM staff will contact you if anything needs to be revised before copyediting and production can begin. Otherwise, you will be notified when your proofs are ready to be viewed.

Data Availability: ASM policy requires that data be available to the public upon online posting of the article, so please verify all links to sequence records, if present, and make sure that each number retrieves the full record of the data. If a new accession number is not linked or a link is broken, provide production staff with the correct URL for the record. If the accession numbers for new data are not publicly accessible before the expected online posting of the article, publication may be delayed; please contact ASM production staff immediately with the expected release date.
- Editor's Note: Bioproject PRJNA1439424 was not public at the time of review.

Publication Fees: For information on publication fees and which article types have charges, please visit our website. We have partnered with Copyright Clearance Center (CCC) to collect author charges. If fees apply to your paper, you will receive a message from no-reply@copyright.com with further instructions. For questions related to paying charges through RightsLink, please contact CCC at ASM_Support@copyright.com or toll free at +1-877-622-5543. CCC makes every attempt to respond to all emails within 24 hours.

ASM Membership: Corresponding authors may join or renew ASM membership to obtain discounts on publication fees. Need to upgrade your membership level? Please contact Customer Service at Service@asmusa.org.

Embargo Policy: A press release may be issued as soon as the manuscript is posted on the mSystems Latest Articles webpage. The corresponding author will receive an email with the subject line "ASM Journals Author Services Notification" when the article is available online.

Cover Image Submissions: If you would like to submit a potential Cover Image, please email a file and a short legend to msystems@asmusa.org. Please note that we can only consider images that (i) the authors created or own and (ii) have not been previously published. By submitting, you agree that the image can be used under the same terms as the published article. Image File requirements: TIF/EPS, 7.5 inches wide by 8.25 inches tall (at least 2,250 pixels wide by 2,475 pixels tall), minimum 300 dpi resolution (600 dpi preferred), RGB, and no figure elements, e.g., arrows or panel labels. The legend should be a short description of the image, 1-2 sentences recommended. Please use the ASM Journals Cover Image Sizing Tool (https://journals.asm.org/pb-assets/html/asm-cover-sizing-tool.html) to ensure that your proposed cover image meets our size requirements.

Thank you for submitting your paper to mSystems.

Sincerely,
Mark Mandel
Editor
mSystems
